# The soot of all evil

**DOI:** 10.7554/eLife.11709

**Published:** 2015-10-21

**Authors:** Derek W Russell, J Edwin Blalock

**Affiliations:** Department of Medicine, University of Alabama at Birmingham, Birmingham, United States; Department of Medicine, University of Alabama at Birmingham, Birmingham, United Statesblalock@uab.edu

**Keywords:** inflammation, Th17, emphysema, human, mouse

## Abstract

Nanoparticles of carbon black in cigarette smoke trigger inflammation in the lung.

**Related research article** You R, Lu W, Shan M, Berlin JM, Samuel ELG, Marcano DC, Sun Z, Sikkema WKA, Yuan X, Song L, Hendrix AY, Tour JM, Corry D, Kheradmand F. 2015. Nanoparticulate carbon black in cigarette smoke induces DNA cleavage and Th17-mediated emphysema. *eLife* **4**:e09623. doi: 10.7554/eLife.09623

**Image** Carbon particles (indicated by red arrow) accumulate in immune cells

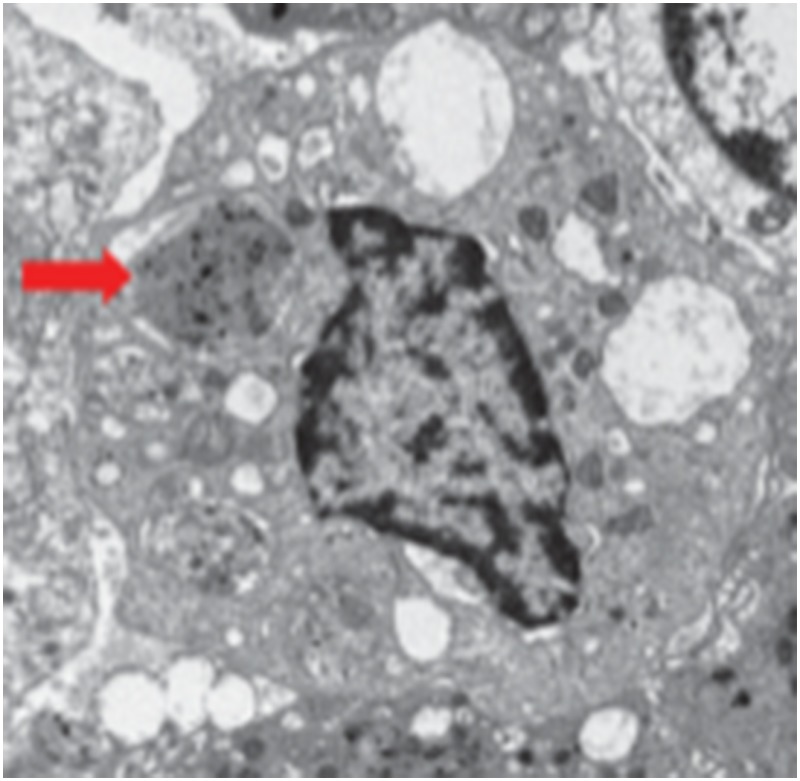

It is common knowledge that a black pigmented material in cigarette smoke (commonly called ‘tar’) stains the human lung. While the ugly images of black lungs on public health advertisements illustrate the damage that can be done by smoking and other sources of inhaled pollution, it was not previously known whether this dark pigment itself was harmful.

Many chemical components of smoke have been connected with tissue damage that can lead to chronic obstructive pulmonary disease (COPD) and other diseases that affect lung function. However, the particles of carbon that dye the lung black have received relatively little attention from researchers because they appear to be rapidly taken up by immune cells, are relatively insoluble, and seem rather inert. Now, in *eLife*, Farrah Kheradmand, David Corry, James Tour and colleagues—including Ran You as first author—reveal important mechanisms by which these particles are able to trigger and sustain a permanent inflammatory process in the lungs (You et al., 2015).

The symptoms of COPD—which is a major cause of suffering and death worldwide (Raherison and Girodet, 2009)—result from the loss of air-sacs (alveoli) and from inflammation in the windpipe and smaller airways of the lung. These processes are driven by local inflammation that leads to the release of enzymes that digest the thin connective tissue between alveoli in a process called emphysema (Djekic et al., 2009). This decreases the surface area over which oxygen and carbon dioxide can be exchanged, and it also compromises the ‘springiness’ of the lungs. As the lungs become floppier and less springy, the airways inside them (themselves already inflamed by the smoke) become more prone to collapse, which makes it difficult for the patient to blow air back out of the lung. As this process progresses, patients develop increasing shortness of breath and respiratory failure.

You et al.—who are based at Baylor College of Medicine, Rice University and City of Hope National Medical Center—first assessed the composition of these black particles. They found that the staining in the lungs of smokers was localized within populations of immune cells called myeloid-derived dendritic cells. These cells consume pathogens and foreign materials in a process called phagocytosis, and then present the digested fragments to other immune cells so that the body can learn to recognize and attack them. You et al. found that the pigment within these cells is composed of clumps of spherical carbon particles: the particles had diameters in the range of 20–50 nanometers, hence the name nanocarbon black. The researchers also found the same material in the equivalent cells of mice that had been exposed to cigarette smoke.

You et al. then studied the effects of nanocarbon black on the mouse lung by administering it through the nose in quantities equivalent to those received by a lifetime smoker (but without the other components of cigarette smoke). This demonstrated that particles of nanocarbon black are sufficient to cause emphysema-like changes in the mouse lungs.

These changes appear to be caused by damage to DNA, which sets off a cascade of effects that culminate in the release of small proteins called interleukin 1β and 6 (Figure 1). Together, these cytokines enable immune cells called T cells to become T helper 17 cells, which promote inflammation and inhibit another type of T cell that normally act to constrain immune responses (Bettelli et al., 2006; Benwell and Lee, 2010). The tendency to cause DNA damage appears to depend on the size of the particles and their solubility in oil: this has important implications both for potential regulations governing the emission of carbon black and for further research on the biological effects of these pollutants.

**Figure 1.**
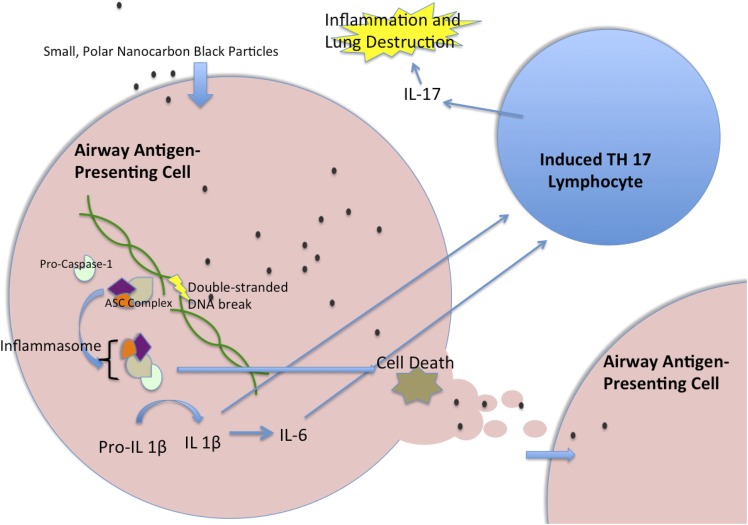
Nanocarbon black triggers perpetual inflammation in the lung. Nanoparticles of carbon black are consumed by antigen-presenting cells, such as human myeloid dendritic cells, where they lead to DNA breaks. This, in turn, initiates the activation of a complex of proteins called the inflammasome, which includes the Caspase-1 protein (Taniguchi and Sagara, 2007). The activated inflammasome leads to the cleavage of Pro-IL1β to make a mature signal protein called IL-1β, while also initiating processes that will lead to the death of the cell. The mature IL-1β causes upregulation of several inflammatory pathways, including the increased transcription of IL-6. These cytokines then act together to trigger T lymphocytes to become T helper 17 (TH17) cells. These cells produce the inflammatory cytokine IL-17, which has been implicated in chronic obstructive pulmonary disease. When the antigen-presenting cell dies, the nanoparticles are released to be taken up by another generation of antigen-presenting cells, which lead to another cycle of inflammation. IL = interleukin; ASC = Adaptor Protein Apoptosis-Associated Speck-Like Protein Containing CARD.

You et al. hypothesize that nanocarbon black is consumed by phagocytic immune cells and then damages the genetic material in these cells so that they die after sending signals to the immune system that promote inflammation. As they die, these cells release their toxic contents, which can then be eaten by another generation of immune cells: this sets up a cycle of inflammation that gradually dissolves connective tissue in the lung.

These findings shed light on an enigmatic question that has troubled medicine for decades: how does cigarette smoke continue to cause lung inflammation and damage years after a patient has stopped smoking? They will also help us to understand the effects of other sources of air pollution and particulates. Since every human on earth is exposed to at least one form of carbon-based pollution, such as wood-burning stoves, petroleum fumes or even electronic cigarette vapor, this research has major implications both for future scientific inquiry and for public health policy.

Further work is needed to characterize the carbon nanoparticles produced by various forms of combustion so that the many public health implications of these findings can be better explored. In the longer term, it may be possible to design new therapies that block the inflammation caused by these particles and slow down the destruction of lung tissue that causes so much suffering in people with emphysema.

## References

[bib1] Benwell RK, Lee DR (2010). Essential and synergistic roles of IL1 and IL6 in human Th17 differentiation directed by TLR ligand-activated dendritic cells. Clinical Immunology.

[bib2] Bettelli E, Carrier Y, Gao W, Korn T, Strom TB, Oukka M, Weiner HL, Kuchroo VK (2006). Reciprocal developmental pathways for the generation of pathogenic effector TH17 and regulatory T cells. Nature.

[bib3] Djekic UV, Gaggar A, Weathington NM (2009). Attacking the multi-tiered proteolytic pathology of COPD: new insights from basic and translational studies. Pharmacology & Therapeutics.

[bib4] Raherison C, Girodet PO (2009). Epidemiology of COPD. European Respiratory Review.

[bib5] Taniguchi S, Sagara J (2007). Regulatory molecules involved in inflammasome formation with special reference to a key mediator protein, ASC. Seminars in Immunopathology.

[bib6] You R, Lu W, Shan M, Berlin JM, Samuel ELG, Marcano DC, Sun Z, Sikkema WKA, Yuan X, Song L, Hendrix AY, Tour JM, Corry D, Kheradmand F (2015). Nanoparticulate carbon black in cigarette smoke induces DNA cleavage and Th17-mediated emphysema. eLife.

